# Supplementary material for: Tonic interferon restricts pathogenic IL-17-driven inflammatory disease via balancing the microbiome
Source: eLife. 2021 Aug 11;10:e68371. doi: 10.7554/eLife.68371 (PMC8376249; doi:10.7554/eLife.68371)
Supplement: Supplementary file 1. [file elife-68371-supp1.docx]

**Supplementary File 1. List of antibodies**
