# Supplementary material for: Tonic interferon restricts pathogenic IL-17-driven inflammatory disease via balancing the microbiome
Source: eLife. 2021 Aug 11;10:e68371. doi: 10.7554/eLife.68371 (PMC8376249; doi:10.7554/eLife.68371)
Supplement: Supplementary file 2. [file elife-68371-supp2.docx]

**Supplementary File 2. Summary for mutant phenotypes**

|  | STAT1 KO | STAT2 KO | GR STAT2 DKO | ARGR DKO | IL-17R STAT1 DKO |
| --- | --- | --- | --- | --- | --- |
| Spenomegaly | Y | Y | Y | N | N |
| Neutrophils expansion | Y | Y | Y | moderate | N |
| Effector T cell increase | Y | Y | Y | N | Y |
| Central memory T cells decrease | Y | Y | Y | Y | Y |
| NK cell decrease | Y | Y | Y | Y | Y |
| Progenitor expansion in spleen | Y | Y | Y | N | N |
| Increase HSC in spleen | Y | marginal | Y | N | N |
| B cell decrease | Y | Y | Y | moderate | modest |
| Increased IFN‑γ production | Y | n.d. | Y | moderate | Y |
| Increased IL-17A production | Y | n.d. | Y | modest | Y |
